# Polycystic ovary syndrome management: a review of the possible amazing role of berberine

**DOI:** 10.1007/s00404-020-05450-4

**Published:** 2020-02-14

**Authors:** M. Rondanelli, Vittoria Infantino, A. Riva, G. Petrangolini, M. A. Faliva, G. Peroni, M. Naso, M. Nichetti, D. Spadaccini, C. Gasparri, S. Perna

**Affiliations:** 1 IRCCS Mondino Foundation, 27100 Pavia, Italy; 2 Department of Public Health, Experimental and Forensic Medicine, University of Pavia, 27100 Pavia, Italy; 3 Department of Biomedical Science and Human Oncology, University of Bari Aldo Moro, 70121 Bari, Italy; 4 Research and Development Unit, Indena, 20146 Milan, Italy; 5 Endocrinology and Nutrition Unit, Azienda Di Servizi Alla Persona ‘‘Istituto Santa Margherita’’, University of Pavia, 27100 Pavia, Italy; 6 Department of Biology, College of Science, University of Bahrain, Sakhir Campus, P. O. Box 32038, Zallaq, Kingdom of Bahrain

**Keywords:** PCOS, Berberine, VAT, Insulin resistance, Nutraceutics

## Abstract

**Purpose:**

The therapy of polycystic ovary syndrome (PCOS) is based on synthetic hormones associated with lifestyle changes, but these therapies cannot be taken continuously, especially by women who would like to become pregnant. Thus, nutraceutical compounds were investigated as possible agents for treatment of PCOS. Berberine is shown to be effective against insulin resistance and obesity, particularly against visceral adipose tissue (VAT). Because of these properties, researchers theorized that berberine could be effective in PCOS treatment.

**Methods:**

The aim of this narrative review was to assess the state of the art about the use of berberine in PCOS management.

**Results:**

This review included 5 eligible studies. Despite the number of studies considered being low, the number of women studied is high (1078) and the results are interesting. Two authors find out that berberine induced a redistribution of adipose tissue, reducing VAT in the absence of weight loss and improved insulin sensitivity, quite like metformin. One author demonstrated that berberine improved the lipid pattern. Moreover, three authors demonstrated that berberine improved insulin resistance in theca cells with an improvement of the ovulation rate per cycle, so berberine is also effective on fertility and live birth rates.

**Conclusions:**

Finally, berberine is safe to use in premenopausal women who want to get pregnant and showed few side effects in all the cited studies. In conclusion, the use of berberine for PCOS is safe and promising, even if more studies are needed to create a consensus about the dosage of berberine useful for long-term therapy.

## Purpose

About 5–10% of pre-menopausal women are affected by Polycystic Ovary Syndrome (PCOS) [1–4] and its treatment could be very long, but always symptom-oriented, and also dynamic and adapted to the changing circumstances and personal needs of the individual patient. Ovarian dysfunction causes metabolic disorders and hyperandrogenism; these should be counteracted by all therapeutic approaches usually used in the management of PCOS [4].

In addition to pharmacological molecules, to improve the metabolic status of women with PCOS, it is highly recommended to suggest a modification of the lifestyle and weight reduction. The goal of all these interventions is the improving of ovulation regularity and the protection against cardiovascular diseases. The clinical treatment of PCOS is often a long-term therapy, and the most commonly used drugs are combined oral contraceptives (COCs), antiandrogenic progestins, and insulin-sensitizing drugs. Anyway, these drugs could treat the respective single sign of PCOS [5].

Because of the length of the therapy that can last for the whole fertile period of a woman and, therefore, could interfere with a hypothetical pregnancy, also botanicals have been considered against PCOS. In particular, berberine (BBR) is an alkaloid plant extract widely used in Chinese Herbal Medicine against infections, hypercholesterolemia, Diabetes type 2 and cancer [6].

Berberine is also shown to be effective against insulin resistance and obesity, particularly against visceral adipose tissue in vitro and in murine models [2, 7, 8].

Berberine is a very promising botanical compound because of the few minor side effects that could present and because its target is an AMP-activated protein kinase (AMPK) common to fatty acid oxidation, glucose generation and insulin resistance [9]. Zhang et al. explained the berberine mechanism of action and after that, scientists began to hypothesize and therefore to use berberine in clinical management of dyslipidemia, diabetes type 2 and obesity in order to counteract the cardiovascular risk derived from these metabolic disorders [9–12]. Moreover, thanks to the extreme tolerance and very minor side effects on long-term treatment, the berberine is the only botanical compound included in European guidelines for the management of dyslipidemia and it is used also in patients who do not tolerate statins [13–15].

Berberine, if associated with a healthy lifestyle, improves women’s body composition and causes androgen’s reduction as pointed out by Saleem et al*.* [3, 8].

PCOS is an endocrine–metabolic disorder very similar to the metabolic syndrome, indeed they have a common factor: insulin resistance. Ong et al. [6] explained that insuline resistance is the key factor that could cause obesity and anovulatory cycles and actually, that should be the target of therapeutic molecules against PCOS and also Metabolic Syndrome [2, 6].

Botanical compounds are always used in traditional Chinese medicine; among those, the berberine induces an amelioration of insulin resistance if administered at a dose of 500 mg per os twice a day for 6 months, and also improves the regularity of menstrual cycle [6].

Berberine is not only used in China, but also is being administered by English medical doctors dealing with women affected by PCOS to counteract PCOS’ symptoms and signs [16]. Rooney and Pendry in their survey among English clinicians pointed out the lack of scientific literature regarding the use of herbal medicine and consequently, the lack of uniformity in herbal prescription against PCOS [16].

Given this background, the aim of the present narrative review was to assess the state of the art about the use of berberine in the management of PCOS.

## Methods

**Fig. 1:**
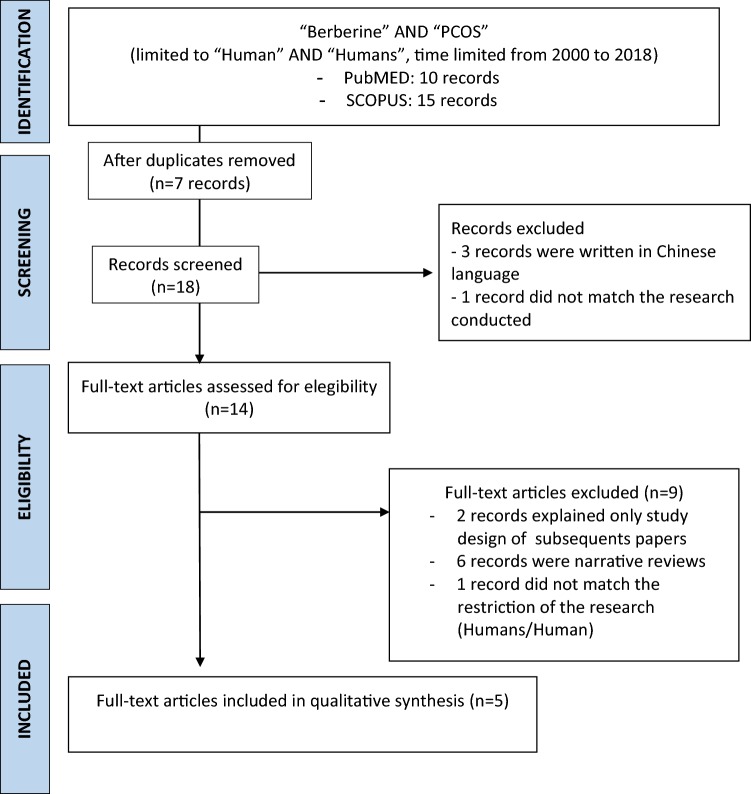
Flow chart of literature research

This narrative review was written after a PubMed and SCOPUS research performed with these keywords: “Berberine”, “PCOS” with the use of Boolean AND operator to establish the logical relation between them (Fig. 1). The research was conducted by skilled operators from July to September 2018 and following Egger’s criteria for systematic reviews [17, 18]. The research was time limited (from 2000 to 2018) and restricted for Human AND/OR Humans studies to know about the state of the art of the use of berberine in women affected by PCOS diagnosed by Rotterdam criteria.

## Results

Only five full-text papers were taken in analysis, and all of them mentioned the possible use of berberine in non-pharmacological treatment of the endocrine disorder. The results of these five studies have been shown in Table 1.

**Table 1:** Trials conducted on the effect of berberine on PCOS-premenopausal women

First author, year (References)	Setting	Number of subject (only F)	Inclusion criteria	Type of intervention	Control group	Intervention group 1	Intervention group 2	Duration	Changes in intervention group/s	Changes in control group	Conclusions	Study design (evidence level)
Wei (2012) [19]	Clinical Centre of Reproductive Medicine (Harbin, China)	100 subjects enrolled (11 lost to follow up or left the protocol)	PCOS (Rotterdam Criteria) and insulin resistance (HOMA-IR > 3.8 or FGIR < 4.5)	All groups received nutritional instruction to limit fat and carbohydrate intake + physical exercise (30 min/day). Control group: placebo + CPA Intervention group 1: BBR hydrochloride + CPA Intervention group 2: MET + CPA	*N* = 28 (age = 26.75 + 2.62). EE + CPA 35 mcg + 2 mg/day	*N* = 31 (age = 25.74 + 2.66) BBR hydrochloride + CPA 500 mg + 2 mg × 3 times/day	*N* = 30 (age = 26.03 + 2.82) MET + CPA 500 mg + 2 mg × 3 times/day	3 months	↓WC, WHR (*p* < 0.01) BBR vs MET. ↓FPG, FIN, HOMA-IR, FGIR, TC, LDL, FAI, TT (*p* < 0.05) BBR vs MET/placebo. ↑HDL,SHBG (*p* < 0.05) BBR vs MET	↓WC, WHR, FPG, FIN, HOMA-IR, FGIR, TC, LDL, TT, FAI non statistically significant between groups. ↑HDL, SHBG non statistically significant between groups	BBR is non toxic compound and shows similar metabolic effects compared to MET	RCT double-blind (level 1b)
Orio (2013) [25]	Salerno University Hospital	100 subject enrolled	PCOS (Rotterdam Criteria) and obesity	No lifestyle modification during intervention. Administration of Berberol™ (PharmExtracta, Pontenure, Italy): 588 mg *Berberis aristata* titered as 85% and 105 mg of *Silybum marianum* titered < 60% 1 tablet × 2 times/day	*N* = 50 obese women (no intervention) (age = 24.12 + 3.6) (BMI = 31.8 + 1.4)	*N* = 50 PCOS and obese women (age = 25.0 + 3.5) (BMI = 32.8 + 2.4)	–	6 months	↓HOMA-IR, AUC_INS_, TC, LDL, TRG, TT, androstenedione (*p* < 0.01 within group) ↑SHBG (*p* < 0.01 within group)	Control group show an IR and hormonal profiles better than PCOS + obese women either first (*p* < 0.01) and after (*p* < 0.01) BBR intervention for all parameters considered	BBR improves metabolic and hormonal profile in PCOS women	Prospective CT (level 2b)
Cicero (2014) [21]	Internal Medicine, Aging and Kidney disease Department, University of Bologna	84 outpatients subjects enrolled	Age from 18 to 45 years; LDL > 130 mg/dL after oral oestroprogestins therapy against PCOS or for pure contraception	All groups: 3-month lipid-lowering diet before the intervention study, then 3-month nutraceutical intervention with BBR 500 mg/tab/day + monacolins 3 mg/tab/day	Non PCOS patients *N* = 40 (age = 29.3 + 5.6)	PCOS patients *N* = 44 (age = 26.5 + 6)	–	3 months + 3 months	↓PCR (*p* = 0.024) between groups after nutraceutical intervention	–	There are not changes in HOMA-IR, nor FPG or HDL	Prospective CT (level 2b)
An (2014) [22]	Clinical Centre of Reproductive Medicine (Harbin, China)	150 subjects enrolled (128 completed the study)	PCOS (Rotterdam Criteria) with previous unsuccessfully IVF treatment	Treatment with -placebo: tablets similar to BBR and MET. -BBR: 500 mg × 3 times/day. -MET: 500 mg × 3 times/day. All groups: after 3 months undergoing ovarian stimulation	Placebo group *N* = 43 (age = 28.4 + 4)	MET group *N* = 41 (age = 28.7 + 4.2)	BBR group *N* = 44 (age = 28.3 + 3.8)	12 weeks	↓ BMI, TC, LDL (*p* < 0.05) BBR vs MET/placebo ↓ WC, WHR, TT, SHBG, FAI, FPG, FIN, HOMA-IR (*p* < 0.05) BBR/MET vs placebo ↑Live birth (*p* < 0.05) BBR vs MET/placebo	//	Berberine is safety to use in premenopausal women and improves, more than metformin, metabolic profile and the respondence to ovarian stimulation and finally the percentage of live birth in PCOS women undergone to IVF	RCT double-blind (level 1b)
Wu (2016) [24]	China (19 hospitals)	644 subjects enrolled	Age from 20 to 40 years; PCOS (Rotterdam Criteria); at least one open fallopian tube and normal uterine cavity; male partner with normal sperm concentration and motility; 1 year of infertility	BBR or BBR placebo: 1.5 mg/tab/day. Letrozole or letrozole placebo: 2.5 mg/tab/day for 3 months, then 5 mg/tab/day	Letrozole group (letrozole + BBR placebo) *N* = 215 (age = 27.8 + 3.6)	BBR group (letrozole placebo + BBR) *N* = 214 (age = 27.8 + 3.7)	Combination group (letrozole + berberine) *N* = 215 (age = 27.8 + 3.6)	6 months	↓BMI (*p* < 0.01), WC (*p* = 0.05) within BBR group. ↑Ovulation (*p* < 0,01) BBR group vs. Letrozole group	↑ Live births (*p* < 0.05) Letrozole group and Combination group vs. BBR group	Berberine improves metabolic profile in PCOS women and not affects the improvement of live birth rates achieved by letrozole	Multicenter RCT double-blind (level 1b)

*HOMA-IR* homeostasis model assessment-insulin resistance, *FGIR* fasting glucose insulin ratio, *EE* ethinyl estradiol, *CPA* cyproterone acetate, *BBR* berberine, *MET* metformin, *WC* waist circumference, *WHR* waist to hip ratio, *FPG* fasting glucose; *FIN* fasting insulin, *TC* total cholesterol, *LDL* low density lipoprotein cholesterol, *FAI* free androgen index, *HDL* high density lipoprotein cholesterol, *SHBG* sex hormone-binding globulin, *TT* total testosteron, *TRG* triglycerides, *IVF* in vitro fertilization;, *BMI* body mass index, *AUC*_*INS*_ area under the curve-insulin, *RCT* randomized clinical trial, *CT* clinical trial

All studies conducted compared berberine with other PCOS pharmacological therapies such as combined oral contraceptives, anti-androgen drugs, metformin and monacolins to counteract cardiac risk in PCOS [19–24].

Authors found that berberine induced a redistribution of adipose tissue, reducing visceral fat mass even in the absence of weight loss, and insulin sensitivity was improved similar to metformin [19, 22]. The nutraceutical compound also improved the lipid pattern in subjects with PCOS against monacolins [21]. Berberine in women with PCOS also improved the insulin resistance in theca cells, thanks to an increase of the expression of Glut-4 in ovaries with an improvement of the ovulation rate per cycle; so, berberine is also effective on fertility and live birth rates in women affected by PCOS [23, 24].

Berberine is safe to use in premenopausal women who want to become pregnant and showed few side effects in all the cited studies, in particular constipation and nausea [23, 24]. Regarding specifically side effects, 3 out of 31 subjects (BBR group) complained of a bitter taste in their mouth versus 9 out of 30 subjects (MET group) suffered by nausea, vomiting, mild diarrhea and flatulence [19]. No adverse events were observed by Cicero et al. [21] and Orio et al. [25], while An et al. reported 1/50 subject (BBR group) transient gastrointestinal side effects including diarrhea vs. 2/50 subjects (Met group) gastrointestinal side effects including diarrhea [22]. Wu et al. reported that 1 subject out of 214 subject (BBR group) had constipation and nausea, no fetal abnormalities in BBR group versus 1 fetal abnormality in letrozole group resulting in termination of pregnancy [24]. All these observed side effects were transient and mild, underlining the good safety of the use of BBR in PCOS patients.

The five studies analyzed showed some differences in the design of the study and in the results; in particular, Wei et al. An et al. and Wu et al. performed their research comparing BBR vs. ethynyl estradiol + cyproterone acetate/ metformin/letrozole, respectively (see details in Table 1). Studies have not been conducted comparing berberine with a real placebo, perhaps because of the studied target population: women of child-bearing age, so it would be probably unethical the administration of real placebo [19, 22, 24]. Only An and colleagues used a real placebo but the placebo group also underwent to ovarian stimulation like the interventions groups to become pregnant [22].

Women enrolled by Wei et al. and An et al. received lifestyle and nutritional counseling before the treatment with berberine, metformin or placebo [19, 22]; while in the study conducted by Orio et al., no lifestyle modifications were implemented [25] in spite of the fact that a good lifestyle has been demonstrated to be crucial to the amelioration of symptoms related to PCOS [3]. Only Cicero et al. before the beginning of the study gave a three-month lipid-lowering diet to enrolled women [21].

Only Wei et al. pointed out a statistically significant reduction of the waist circumference, the waist–hip ratio and the level of sex hormone-binding globulin in the berberine group vs. the metformin group (*p* > 0.05), and this result is not confirmed by other research groups [19]. An et al. showed a decreased BMI in the group of berberine vs. metformin (*p* < 0.05) [22].

Women affected by PCOS showed an impaired fasting glucose control and insulin resistance, and this aspect was investigated by all authors except Wu and colleagues [24]. Parameters taken into consideration from the authors were fasting glucose, fasting insulin, the HOMA index and the AUC_INS_; in all studies, berberine showed an improvement of the parameters with a reduction of insulin resistance in a statistically significant way only in berberine groups vs. placebo (*p* < 0.01 or *p* < 0.05), so berberine reduced insulin resistance but is not superior to metformin [19, 21, 22, 25]. All these authors also analyzed changes in the lipid profiles of PCOS women, and demonstrated that berberine improved, in all patients, HDL cholesterol and reduced total cholesterol and LDL cholesterol [19, 21, 22, 25].

Only Cicero and colleagues have studied the effect of berberine on the Protein C Reactive (PCR) and on inflammation [21]. Cicero et al. found out that berberine in obese PCOS women causes a reduction of PCR statistically relevant compared to obese subjects (*p* = 0.024) [21].

Only two research groups focused on the effect of berberine on pregnancy and live births in PCOS women [22, 24]. An et al. focused on live birth percentages and showed that the berberine group had a percentage of live births greater than the metformin group (*p* < 0.05) and the placebo group (*p* < 0.05) [22]. Wu et al. confirmed the positive association between berberine treatment and live births in PCOS women, with odds ratio higher than 1 in comparison between the berberine group vs. placebo (*p* = 0.004) and the berberine group vs. letrozole group (*p* = 0.001) [24].

All research groups had analyzed the effect of berberine on women affected by PCOS, but nutraceutical and drugs formulations used were quite different; in particular, Wei et al. and An et al. administered berberine hydrochloride 500 mg three times/day [19, 22], and Orio et al. administered a tablet twice a day, with 588 mg of *Berberis aristata* extract tittered as 85% of berberine [25]. Cicero et al. tested a nutraceutical compound tablet with 500 mg of berberine plus 3 mg of monacolins [21]; Wu et al. administered berberine in a daily dose of 1.5 g [24]. There was a great difference between the extracts of berberine used in these five studies, and only in one case, the extract of berberine was tittered, but not standardized. This paper pointed out the lack of literature about the use of berberine in the treatment of PCOS, and shows the need to conduct more clinical trials on this topic to create a consensus regarding the amount of berberine useful in creating positive effects in PCOS women in a long-term therapy.

## Conclusions

Despite the number of studies taken into consideration in this review being low, the number of women considered in these 5 studies is high (1078), and the results of these studies are interesting.

Two authors found out that berberine induced a redistribution of adipose tissue, reducing visceral fat mass even in the absence of weight loss, and insulin sensitivity was improved similar to metformin. One author demonstrated that berberine improved the lipid pattern. Moreover, three authors demonstrated that berberine improved the insulin resistance in theca cells with an improvement of the ovulation rate per cycle; so berberine is also effective on fertility and live birth rates. Finally, berberine may be considered as a safe botanical compound to use in premenopausal women who want to become pregnant, because of the few side effects showed in the five reported studies. Furthermore, scientific literature provides a large number of studies which reported safety and tolerability on long-term treatment with berberine in humans. In conclusion, the use of berberine in women with PCOS is very promising, even if more clinical studies are needed to confirm the safety and the efficacy of the berberine associated with other pharmacological compounds used in long-term therapy of PCOS. Besides further studies are needed to create a consensus regarding the dosage of berberine useful to create positive effects in PCOS women, even in long-term therapy.
